# People living in nursing care facilities who are ambulant and fracture their hips: description of usual care and an alternative rehabilitation pathway

**DOI:** 10.1186/s12877-019-1321-x

**Published:** 2020-04-09

**Authors:** Maggie Killington, Owen Davies, Maria Crotty, Rhiannon Crane, Naomi Pratt, Kylie Mills, Arabella McInnes, Susan Kurrle, Ian D. Cameron

**Affiliations:** 1 Flinders University, South Australia, Australia; 2 Southern Adelaide Local Health Network, South Australia, Australia; 3 Cognitive Decline Partnership Centre, University of Sydney, Hornsby Ku-ring-gai Hospital, Hornsby, New South Wales Australia; 4 John Walsh Centre for Rehabilitation Research, Kolling Institute, Faculty of Medicine and Health, University of Sydney, Camperdown, Australia

**Keywords:** Hip fracture, Nursing home, Nursing care facility (NCF), Usual care, General practitioner, Physical therapy, Dietetics, Rehabilitation, Aged care, Falls

## Abstract

**Background:**

Little is known about treatment provided to people living in nursing care facilities (NCFs) after hospital admission for hip fracture. In addition, there are no clinical guidelines for rehabilitation and recovery following hip fracture for nursing home residents.

**Methods:**

As part of a randomised trial (SACRED trial), which investigated the efficacy of a four week in-reach rehabilitation program, data were collected which described routine care for 240 people living in 76 nursing care facilities in South Australia who fractured their hips. The in-reach rehabilitation provided to 119 intervention participants is described, including intensity, type and methods used to encourage participation in rehabilitation. Adverse events that occurred, in particular falls, are also reported.

**Results:**

NCF records indicated that, over the four weeks following discharge from hospital after hip fracture, 76% of patients receiving usual care had a consultation with their general practitioner. Physiotherapy was provided to 79% of patients in usual care (median of 1.96 h over the 4 weeks, which is less than 30 min each week of physiotherapy). In-reach rehabilitation was provided by the hospital team for 13 h over the 4 weeks with almost full attendance at physiotherapy sessions (median of 1 missed session, range 0–7 with a median of 14 physiotherapy sessions attended by participants, range 1–18). Experienced therapists provided a flexible approach to the rehabilitation to account for patients’ dementia and associated neuropsychiatric symptoms while providing dietetic support, mobility training and education to nursing home staff. The number of falls experienced by those in the intervention group was higher compared to those in usual care (Relative Risk 1.38 (95%CI 1.04–1.84, *p* = 0.03).

**Conclusions:**

Rehabilitation can be provided to people living in NCFs following hip fracture, even when they have moderate to severe dementia but the model needs to be flexible. Provision of rehabilitation may increase the rate of falls in this population. Further studies are required to establish the feasibility of the intervention in other long term care settings. (327 words).

**Trial registration:**

ACTRN12612000112864 registered on the Australian and New Zealand Clinical Trials Registry (ANZCTR).

## Background

People living in nursing care facilities (NCFs) are often excluded from participating in research and clinical trials [1] which has contributed to a lack of information regarding the usual clinical care provided in NCFs to residents following their return from hospital after hip fracture surgery. This lack of information makes the development of practice guidelines difficult. While there is evidence suggesting that rehabilitation following hip fracture repair improves outcomes and that this rehabilitation is best provided as part of an organised multidisciplinary health care team it is unclear how services should be provided to people living in NCFs, the majority of whom have dementia [2, 3]. There are, as yet, few protocols to guide treating therapists who work with residents living in NCFs after their return home. Little is known about what types of therapy and how much therapy people who live in residential aged care are receiving following a hip fracture. It is also unclear whether they will tolerate team based rehabilitation approaches.

The SACRED trial (ACTRN12612000112864 registered on the Australian and New Zealand Clinical Trials Registry (ANZCTR)) recruited 240 older people from NCFs who were previously mobile and had fractured their hips and randomly allocated them to receive a 4 week in-reach geriatric rehabilitation program or usual care on discharge [4]. This study involved very old people (mean age 88.6 years (SD 5.6)) most of whom suffered with dementia. Only 2 people in the study had a Mini-Mental State Examination (MMSE) score over 25 and could provide their own consent. We used the data from this study to describe usual medical and therapy practice in nursing homes and to outline an alternative rehabilitation approach.

Using data from a randomised controlled trial examining a 4 week model of in-reach rehabilitation to people who had fractured hips and were living in nursing homes we aimed to:

1. Describe usual therapy and medical care provided to nursing home residents in the first four weeks after return from hospital following hip fracture surgery.
2. Provide an overview (including participation rates) of an alternative rehabilitation approach provided to those participants randomised to the intervention arm of the SACRED trial.

## Methods

As part of the SACRED trial, data were collected describing routine care provided in the 76 NCFs participants returned to following their surgical hip fracture repair. In addition, data regarding the in-reach rehabilitation provided to those NCF residents allocated to the intervention group, were collected. Ethics approval was gained from Southern Adelaide Clinical Health Research Ethics Committee (8th June 2012 (020.12)). Written consent was obtained from participants’ next of kin.

The data used to inform the current paper include demographic descriptors of the participants and the NCFs where the participants lived, as well as the routine clinical care provided to people living in the NCFs by facility staff and visiting clinicians following hip fracture. In addition, we describe the in-reach rehabilitation received by participants allocated to the intervention arm of the SACRED trial, including adverse events during the first 4 weeks after their return home.

### Data collection

Following discharge from hospital, trial participants were followed up 4 weeks after returning to their NCF to describe what services had been provided.

1. *Usual Care.* An un-blinded research assistant met with staff of the NCF to complete an audit of health related input received by each participant as part of their usual care over four weeks starting at time of their discharge home from hospital. We included any appointments which were health related or required as a consequence of the hip fracture.
2. *The Therapeutic Environment Screening Survey for Nursing Homes (TESS*) which described the accommodation and care environment was completed by the blinded outcome assessor for each participant.
3. *In-reach rehabilitation*. The intervention was quantified in terms of number of sessions, missed sessions and duration of therapy. Treating rehabilitation clinicians kept detailed records of the type of interventions provided. Interdisciplinary team meetings were held weekly to discuss each participant who was receiving intervention and to develop and review approaches. These weekly meetings were led by the trial coordinator, who is an experienced neuro-physiotherapist who has worked in brain injury rehabilitation for more than 30 years. Clinical progress was documented in each patients’ clinical notes. The treating clinicians, in addition to their experience working with patients after hip fracture, regularly worked with people with neurological conditions, including brain injury, stroke and progressive neurological disorders. The physiotherapist who delivered most of the intervention program worked with patients with brain injury several days a week. The treating team adopted many approaches shown to be efficacious in brain injury rehabilitation due to a lack of clinical guidelines for this cohort with advanced dementia. Strategies used included motivational interviewing, plus learning and coaching models shown to be useful in populations with acquired brain injury. At the completion of the trial the clinical team met in a focus group to discuss and authenticate approaches used and, with the assistance of clinical notes and videos of treatment sessions, to summarise which methods had been used throughout the trial to support participant engagement.
4. *Adverse events.* Data were collected on adverse events (including mortality, falls and medical events) recorded in the residents’ clinical notes following their return from hospital by an un-blinded research assistant.

### Data analyses

Data was summarised using descriptive statistics to describe interventions for the usual care and in-reach rehabilitation participants and adverse events for all participants. Relative risk of falls for the intervention group was calculated by dividing the cumulative incidence of falls in the in-reach rehabilitation group by the cumulative incidence of falls in the usual care group.

Qualitative data collected from weekly patient discussions and the focus group held with clinicians were summarised in note form, read a number of times by MK and organised topically to attempt to categorise approaches that had been used by the clinicians to encourage engagement in rehabilitation. A follow-up meeting was subsequently held with clinicians (RC, NP and AMc) to discuss the categories, until consensus had been reached.

## Results

### Participants

The 240 participants resided in 76 different NCFs in the southern, central suburbs and southern peri-urban areas of South Australia spanning 95 km from north to south. Of the 240 participants, 186 (77.5%) had a pre-existing diagnosis of dementia with 73 residents living in dementia care units. On discharge from hospital, 10.8% received “low level care” with 87.5% receiving “high level care”, including Dementia Care (*n* = 73) and Aging in Place (*n* = 35) (see Table 1).

**Table 1:** Accommodation type on return to NCF after surgical repair

TYPE OF UNIT (*n* = 240)	
Dementia special care unit (segregated)	46
Dementia special care unit (cluster)	13
Non special care dementia unit	14
High level care	102
Low level care	26
Ageing in Place	35
Rehabilitation hospital	1
Withdrawn	2
Missing data	1

Participants randomised to the usual care and intervention groups had a mean Mini Mental State Examination (MMSE) score of 8.5 (SD 7.6) and 7.5 (SD 8.0) out of 30 respectively.

### Usual care

All three hospital recruitment sites had an ortho-geriatrics service that provided comprehensive geriatric management during the participants’ hospital admission. On return to the NCF, residents received medical care from a general practitioner and all nursing home sites had contracts with physiotherapists or occupational therapists.

NCF records indicated that 76 and 84% of residents allocated to usual care and in-reach rehabilitation had a consultation with their general practitioner in the first 4 weeks after their return home (see Table 2). In addition, NCF physiotherapists provided services to 78.5% (usual care) and 73% (in-reach rehabilitation) residents, while dietetic consults were sought for a minority of residents (12.4% in usual care group and 8.4% in the in-reach rehabilitation group). See Additional file1: Table S1 for additional usual care received.

**Table 2:** Usual Care medical, physiotherapy and dietetics (four weeks) retrieved from nursing home staff according to randomisation allocation

Health Service	Usual Care (*n* = 121)	In reach rehabilitation (*n* = 119)
*General Practitioner*		
Number of patients who received service	92 (76%)	100 (84%)
Occasions of service		
Mean (SD)	3.25 (1.75)	3.29 (2.31)
Median	3	3
Range	1 to 7	1 to 13
Total time in consultation (mins)		
Mean (SD)	37.47 (26.48)	35.20 (23.38)
Median	30	30
Range	10 to 160	10 to 130
*Physiotherapy*		
Number of patients who received service	95 (78.5%)	87 (73%)
Occasions of service		
Mean (SD)	7.02 (4.62)	3.74 (3.35)
Median	6	3
Range	1 to 25	1 to 18
Total time in consultation (mins)		
Mean (SD)	140.65 (116.59)	70 (62.77)
Median	117.5	47.5
Range	3 to 880	10 to 315
Number of patients prescribed equipment	72	59
*Dietician*		
Number of patients who received service	15 (12.4%)	10 (8.4%)
Occasions of service		
Mean (SD)	1.33 (0.62)	1.5 (0.85)
Median	1	1
Range	1 to 3	1 to 3
Total time in consultation (mins)		
Mean (SD)	27.33 (14.13)	26 (15.6)
Median	20	20
Range	15 to 60	10 to 60

### In-reach rehabilitation

#### Model of care

All the therapists who provided intervention to the trial participants worked as part of the home rehabilitation team of a major rehabilitation hospital. The three key disciplines involved were medical (geriatrician and ortho-geriatric registrars), physiotherapy (with assistance as required from therapy assistants) and dietetics. Other disciplines referred to as necessary included rehabilitation nursing and speech pathology (see Table 3 and Additional file 1: Table S2).

**Table 3:** Occasions of service and duration of in-reach medical, physiotherapy and dietetics

Rehabilitation provided (hours)	
Mean (SD)	12.99 (3.29)
Median (IQR)	13 (11.6–14.8)
Range	2–21.20
**Medical**	
*Geriatric review provided*	114
Deceased	3
Missing data	2
Received 1 medical review only	110
Received more than one medical review	4
*Time to geriatric review* (days) following discharge home	
Mean (SD)	3.7 (3.9)
Median	3
Range	0–26
*Family meeting provided*	110
Face to face in person at nursing home	96
Teleconference	14
*Time to family meeting* (days) following discharge home	
Mean (SD)	11.3 (7.6)
Median	9.5
Range	0–35
*Family meeting not provided*	9
Family unable	1
Deceased prior to family meeting	3
Hospital admission prior to family meeting	1
Missing data	4
**Physiotherapy**	
*Received physiotherapy*	119
Number of visits	
Mean (SD)	13.71 (3.29)
Median	14
Range	1–18
*Time spent in therapy*(hrs)	
Mean (SD)	10.65 (3.16)
Median	10.75
Range	1–18.67
Number of *missed sessions*	
Mean (SD)	1.07 (1.52)
Median	1
Range	0–7
**Dietetics**	
*Received initial DT review*	107
Face to face	107
*Received follow-up DT review*	90
Face to face	3
Phone review	87
*Receive further follow-up review*	5
Face to face	1
Phone review	4

Participants allocated to the in-reach rehabilitation received a median of 13 h of rehabilitation in total over 4 weeks, excluding travel time. NCF residents were seen on the day of discharge or the following day in the nursing home by the in-reach physiotherapist and received a median of 14 visits and 10.75 h of therapy over 4 weeks (see Table 3). Patient adherence was high with all participants only missing a median of 1 physiotherapy session (range 0–7).

Each in-reach participant was visited at the NCF within 48 working hours of their return home by the ortho-geriatric registrar. The registrar undertook a health review focusing on medications, pain and co-morbidities. In addition, a formal meeting with families was held with the geriatrician within the first fortnight to discuss progress, provide education and to discuss end of life planning if required. The interventions provided by the ortho-geriatric registrars and geriatrician are detailed in tables below (see Table 3 and Additional file 1: Table S3). In addition, when malnutrition was identified as an issue using a validated screening tool, the dietician would attend the nursing home within the first 48 h.

Clinicians reported that they needed to understand the co-morbidities associated with ageing and frailty as well as the sequelae potentially related to dementia while managing the main diagnosis of a traumatic hip fracture and subsequent repair. The clinicians were familiar with published evidence based hip fracture guidelines [3]. When planning treatment, the therapists used motivational approaches to encourage participation in the rehabilitation process. In addition, they needed to respect the service guidelines of each NCF, in particular in relation to occupational health and safety processes and procedures, which excluded many practices utilised in rehabilitation programs to encourage mobility, including facilitating safely early sit to stand transfers with 2–3 people assisting. Rehabilitation facilities require a 24 h approach with all team members encouraging use of new patient skills amid a service philosophy of supporting acquisition of patient goals. We found that most NCF staff were less willing, and less likely to be permitted, to support practice in between therapy sessions, but instead only supported implementation of an activity once the skill had been fully acquired. Therefore, interventions were adapted to not only each patient according to their clinical needs, but also each facility according to their culture, beliefs and health and safety guidelines. This disparity was seen particularly during mobility retraining. Participants who safely used a stand lifter with the therapist, particularly in the early phase of their treatment were often not able to use the equipment with NCF staff because of fears that the procedure incurred a risk of injury. Although therapists attempted to work closely with NCF staff to increase their knowledge and improve their confidence, participants who were walking in therapy with light to moderate assistance of one person were often considered not yet ready to walk with NCF staff.

The rehabilitation team met weekly to review each patient’s status, and discuss their goals and progress. In addition, the team discussed issues related to engagement in therapy, any expected and unexpected barriers to rehabilitation and different approaches that might facilitate patient improvement. The intervention was guided by a person-centred tactic as recommended by experts who work with people with dementia, including “feeling” each person’s meaning even when verbal communication was difficult or absent [5]. Clinicians liaised with NCF staff and families to attempt to understand each person in the context of their life story, values and beliefs prior to their hip fracture and working with the person instead of “doing” to them.

Therapists worked with the geriatrician as well as the residents’ general practitioners (GPs) and NCF staff to ensure pain medication was provided prior to a scheduled session if required. In addition, sessions were provided at times that suited the resident, and when they were more likely to be receptive to the challenges of rehabilitation.

Many patients were resistive to challenging physical activities such as standing and walking following their hip fracture repair so the therapist would engage with the person to determine whether there was an activity or exercise that was motivating for the patient. Often patients would refuse to stand for formal “exercise” due to fear but would agree to, for example, “go to the toilet”. Physiotherapists used this strategy frequently, especially in the earliest stages of rehabilitation. In this way, therapists utilised the “motivational interviewing” approach, and assisted the patient to undertake an activity important to them. Also, as therapists were working predominately with patients with moderate to severe dementia they utilised their implicit memory system to encourage individuals to undertake procedures that they remembered and valued like walking to the dining room, rather than overwhelming their explicit memory system by direct instruction.

Often patients required extra support and encouragement before they were willing to overcome their fears and, at these times, therapists would spend time developing a therapeutic relationship with them, including attempting to understand how each patient viewed themselves and what roles they valued. In this way, they aimed to understand how they might support each person to work towards regaining their role or identity. This method of “identity remapping” [6] assisted people to overcome feelings of helplessness and find a sense of purpose. This method allowed them to set rehabilitation goals which are essential in rehabilitation.

#### Medical input

The ortho-geriatric registrar reviewed residents after a median of 3 days and a family meeting was organised with the specialist geriatrician in a median of 9.5 days.

The trial geriatrician had worked as a consultant geriatrician at one of the three recruitment sites for 9 years. The ortho-geriatric registrars were physician trainees undertaking advanced training in geriatric medicine (5–7 years post-graduation), working as part of their 6 month ortho-geriatric rotation. Service data collected by trial doctors demonstrated the medical assessment had features of Comprehensive Geriatric Assessment (CGA) and on all occasions included development of a detailed management plan, with discussions relating to end of life planning for 52% of intervention participants (see Additional file 1: Table S3). A written summary of the initial assessment was provided to both the treating GP and the NCF senior nursing staff, with recommendations on pain management and rationalising other medications. A stepwise approach to analgesia was used, in line with standard guidelines [3, 7, 8]. Advice was provided to the treating GP regarding monitoring for side effects and weaning the analgesia as the participant’s pain resolved. Given the risk of constipation with opiate analgesics, advice about prevention of constipation was also provided.

#### Physiotherapy input

Physiotherapy was focused on restoration of transfers and mobility over short distances. Three physiotherapists were involved in providing therapy to those participants enrolled in the in-reach rehabilitation arm of the trial. Each of the physiotherapists had worked for more than 5 years in the community rehabilitation team.

They worked with aged care staff on transfers and mobility and encouraged a rehabilitation approach between formal therapy sessions. Physiotherapy interventions included bed, chair and standing exercises; resisted arm and leg exercises; transfer practice; and mobility training. The physiotherapists worked with the NCF physiotherapists, nurses and carers whenever possible to provide education about recovery from hip fracture in general and more specifically in relation to each participant. The interventions provided by the rehabilitation physiotherapists are detailed in Table 3 and Additional file 1: Table S4.

Formal physiotherapy sessions were provided by the physiotherapist with the assistance of a therapy assistant, who was a member of the in-reach team, when required due to a high behavioural, emotional or physical burden of care (see Additional file 1: Table 4 for type of physiotherapy provided). NCF residents engaged in bed exercises early after their transition home but on the initial visit were also provided a suitable chair to sit in and assisted to get out of bed. Transfer training consisted of practicing stand transfers or transfers with lifter devices when the person could not yet safely stand. The majority of therapy initially had a functional approach utilising activities that were familiar to the participant prior to their hip fracture eg going to toilet, as residents demonstrated fear, pain and resistance when encouraged to move. As the rehabilitation continued over the 4 weeks, 11% of residents began to participate in more formal therapy, for example Otago balance exercises [9] and resistance exercises. The number of people who could participate in these exercises requiring use of their explicit memory system was low, and therefore challenging balance activities were incorporated within functionally oriented therapy. Thirty seven percent of residents engaged in resistance exercises. Attempts to encourage standing, stand transfers and some mobility remained the focus of therapy throughout the 4 weeks of rehabilitation.

In contrast, the control group received on average 118 min of physiotherapy as part of routine care in the 4 weeks following their return home from hospital which equates to just 35 min a week.

#### Dietetic input

The dietetics review usually occurred at the same time as the first physiotherapy review with 107 people receiving a face to face, in person initial assessment and 90 participants receiving follow-up reviews, usually by phone (see Table 3). Two dieticians provided dietetic support to the trial participants and both had between 3 and 5 years’ experience in rehabilitation and orthopaedic settings. Dietician consults included an initial assessment with recommendations about meal set up and feeding assistance as well as appropriate textures and high energy foods prescription. They requested and reviewed patient’s weights and provided a more intensive nutrition intervention when required (see Table 3 and Additional file 1: Table S5). The dieticians focussed on reducing weight loss by encouraging use of a food chart.

#### Other input

When required rehabilitation nurses and speech pathologists visited the facility (see Supplementary Table 2). All team members would contact family and liaise with NCF staff as frequently as required.

### Adverse events

Three hundred and seventy four adverse events were reported for all participants during the 4 weeks following their return home, with 58% (*n* = 217 in 78 participants) reported for those receiving in-reach rehabilitation and 42% (*n* = 157 in 60 participants) for those receiving usual care. These adverse events were able to be classified in four main categories: Falls, Skin tears/wounds, Medical (Chest infection/pneumonia) and Medical (other). Mortality and re-fracture rates data has already been reported in the main study publication [4] . Falls were the most commonly reported adverse event (*n* = 258), with those receiving the addition of in-reach rehabilitation incurring 62.7% of the falls, with 12 people (21%) requiring hospital admission and three people sustaining hip fractures. Of the usual care group who fell, 15 people (38.5%) required a hospital admission with one person sustaining a hip fracture (see Additional file 1: Table S6).

Ten participants allocated to the intervention arm died within 4 weeks of randomisation. The individual participants received rehabilitation for between 1 and 3 weeks until they either died suddenly, or their management transitioned to comfort care after discussion with next of kin in response to a marked decline in health. Seven out of ten participants died in their facility, one person died on the day of transfer to hospital while 2 others spent 3 and 6 days respectively in hospital prior to their death. Most (9/10) died of an acute medical event or complication rather than progression of underlying chronic disease with the death registry reporting mainly respiratory or combined cardiac and respiratory causes of death (see Additional file 1 Table S7).

## Discussion

The key learnings from the program were that clinicians needed to be innovative, flexible and show significant persistence to promote engagement, especially with patients who had advanced dementia. Most of the patients had not received any rehabilitation while in hospital due to their reduced arousal and reluctance to participate, or overt, challenging behaviours and apparent resistance [10]. Not one of the participants undergoing the SACRED rehabilitation had moved away from their bedside after surgery in the acute hospital setting. A patient’s ability to complete early physiotherapy after surgery is a positive predictor of improved mobility following hip fracture [11]. However, the participants in this trial had received minimal physiotherapy in the acute setting, due to their reluctance to engage in therapy, which was an additional barrier to recovery.

In the 4 weeks after return from hospital residents in the usual care group received less than 2 h (118 min) of physiotherapy and those in the intervention group received 13 h of rehabilitation. A Canadian study which sought therapists’ opinion about how much therapy they provided to their residents in long term care following hip fracture repair [12] reported that residents who were within 6 weeks of fracture would be seen typically 5 times a week (50%), 3–4 times a week (40%) and 1–2 times a week (10%) by the physiotherapist and 5 times a week (40%) and 3–4 times a week (60%) by the occupational therapist. This frequency of sessions far exceeds the rehabilitation provided routinely in Australian NCFs.

There is no evidence that patients from nursing home facilities prefer not to receive rehabilitation. A previous study of eighty-seven inpatients (and where appropriate carers) in acute and rehabilitation hospitals were interviewed to assess preferences for rehabilitation following surgery to repair a hip fracture [13]. Overall, participants expressed a strong desire to participate in a rehabilitation program aimed at regaining mobility, and were prepared to endure moderate pain and effort. Importantly, there were no significant differences between the preferences of patients from NCFs and the community, which suggests that patients from NCFs should be provided the same opportunity to participate in rehabilitation as patients from the community [13].

Focus groups with trial clinicians explored which rehabilitation strategies appeared to be most useful when treating this complex cohort of participants who had advanced dementia and were living in an NCF. In addition to optimising pain management, therapists drew on a number of motivational strategies to encourage engagement, as well as using learning techniques that were most likely to support patients with severe dementia. Rather than use any one technique, therapists often drew on a number of learning models which they used simultaneously eg use of errorless learning as part of procedural learning, which involves errorless practice as part of practical routines eg dressing, toileting.

Errorless learning (EL), as opposed to errorful learning (EF) has been shown to be a superior method of teaching people with dementia to acquire meaningful activities and possibly increase their independence and autonomy while reducing carer burden [14]. A systematic review investigating the efficacy and effectiveness of EL in people with dementia indicated that most trials had been undertaken with people with minimal-mild-moderate dementia rather than people with moderate to severe dementia as in our trial. One small study (*n* = 14) has investigated the acquisition of instrumental activities of daily living and showed that EL and learning by modelling (LM) were more efficacious than trial and error learning (TEL) for a group of people with MMSE scores that ranged between 10 and 26 [15]. There is very little research in people with more advanced dementia. One study demonstrated that errorless learning was more effective than errorful learning but that even a short delay of 10 min reduced any difference [16]. However when the combination of an errorless learning approach and repeated exposure to the cueing was provided recall scores improved in Alzheimer’s disease [17]. In this current trial, therapists coached patients by using words that were understandable and meaningful and by repeating similar commands consistently with some success. Other clinicians have used a similar approach with people following an acquired brain injury who have severe memory problems [18].

The Antecedent-Behavior-Consequence-Model suggests that resistiveness to care can be prevented by understanding the antecedents to the behaviour [19]. The paradigm used by clinicians in this current study to deliver rehabilitation assumed that their own behaviour needed to be flexible to remove identified antecedents as much as possible. In particular, a common antecedent to resistiveness in this cohort was pain, and it was important to ensure pain management was optimum and prescribed at the correct time to support residents’ mobility efforts. Therapists worked closely with the geriatrician, ortho-geriatric registrar and resident’s GP so that pain medication was appropriate and provided at regular intervals and, in particular, prior to attempts to mobilise.

Physiotherapists who reported working successfully with people with dementia following hip fracture spent time developing a rapport with their patients by adapting their communication and developing a “person centred care approach” as opposed to a biomedical approach [20]. We found it was important to not explain participants’ behaviour in terms of their personality or disposition which places undue emphasis on internal characteristics of the person rather than searching for external factors. Clinicians needed to manage pain, offer reassurance and hope, be flexible in their approach, stay calm and take responsibility for outcomes. This often involved working with family and NCF staff to develop an individualised approach to discover what was relevant and meaningful to the participant.

Motivation and engagement are core principles for “non-rehabilitation” goal setting, but the required attributes of self-awareness and motivation may be lacking in a population of patients with dementia. There is some evidence to indicate that “identity remapping” may improve engagement following severe acquired brain injury [6] and we found this to be useful with a number of participants in this current study. Reconstruction of a compelling and realistic identity is important to progress rehabilitation [21]. We sought to use the knowledge gained in other populations to attempt to support patients with dementia to gain a sense of self again after return to the nursing home. Identity remapping was found to be helpful at times in this current trial when residents were unable to visualise goals to facilitate their participation and they were supported to resume previous interests or consider new activities as part of the rehabilitation process.

One of the most consistent challenges when treating people with dementia is helping them manage associated behavioural changes including lack of motivation, ambivalence and resistance. In particular, agitation is a common symptom of dementia [22] and clinicians need to encourage participation in rehabilitation while avoiding exacerbating this symptom. Patients may display behaviours that suggest fear and distress as well as symptoms including hallucinations [23].

In addition, people with advanced dementia frequently display sensorimotor deficits, medical co-morbidities and psycho-social issues in combination with their cognitive impairment [24]. They may display in-coordinated movements, increased muscle tone, and reduced postural control and balance as part of their sensorimotor disorder. When these individuals suffer a hip fracture, the clinical team need to manage both the current orthopaedic condition and the complex pre-existing issues.

The in-reach rehabilitation required regular adaptation for many participants when they had severe dementia and had difficulty using their explicit memory system. It has been reported that pain, fear of falling and reluctance to shift weight on the affected leg results in patients spending most of their time in lying or sitting [24] and this was certainly the starting point for the majority of our participants on their return home from hospital. Much time was spent by clinicians setting up bedrooms, providing prophylactic skin care (eg sheepskin booties, pressure relieving mattresses, better nutrition), sourcing suitable chairs, developing transfer techniques, encouraging movement between positions, and initiating sitting and transfer routines in the NCF. When participants could utilise their explicit memory system, and undertake more challenging demands, participants would undertake graduated resistance programs for upper and lower limbs, and balance exercises which required them to attend to their balance in a focused manner and gradually undertake more challenging activities, for example maintain balance during perturbations, manage steps, obstacle courses etc. However, only 37% of participants could tolerate and manage graduated resistance exercises and an even smaller percentage (11%) of participants could engage in standard balance exercises due to their severe dementia. Therapists needed to plan functional activities that would challenge these modalities including carrying objects, kicking objects, and maintaining balance on uneven surfaces. Therapists were required to be innovative, flexible and persistent to ensure all aspects of the rehabilitation phase were addressed within the complexity of dementia and the NCF environment.

Hypothesis testing was commonly used to attempt to understand what factors were barriers to patients’ engagement in therapy and a number of different techniques were trialled to attempt to find the optimum approach for individual patients. Similar to our trial therapists, a group of physiotherapists working in the United Kingdom (UK) identified that treating patients with dementia after hip fracture was challenging, but that it was possible by “thinking outside the box” to realise each person’s potential [20].

A systematic review suggested that people with mild to moderate dementia following hip fracture can show improved function, walking skills and reduced falls risk as a response to rehabilitation [25]. The participants in this current study showed improved mobility at 4 weeks compared to the usual care group; according to the Nursing Home Life Space Diameter, residents who underwent rehabilitation were more likely to venture further within the nursing home and they required less assistance. However, unlike the outcomes of the systematic review, the risk of falls in this current study increased for those who received rehabilitation. It is possible that people with severe dementia are not as vigilant of potential hazards, and fail to self-monitor themselves as well as those with less severe dementia, and this needs to be considered for this cohort.

Full recovery following hip fracture is reported to require three stages of rehabilitation following acute care [26]. Up to three months post-operatively, early post discharge physiotherapy should be focussed on safe mobility, improving muscle function and keeping as active as possible. In fact, those patients who recover better are those who complete a planned physiotherapy program early after surgery prior to going home [11]. This should then be followed by the post-acute phase when the fracture is healed, and include a more intense program focused on balance, functional activities and endurance. And lastly, between 6 and 24 months, improvements have been demonstrated when more challenging activities have been continued, for example resisted activities and more challenging balance therapy. This model is unlikely to be applicable to NCF residents due to their cognitive impairments, limited life expectancy and the unavailability of funding for this intensity of treatment.

Ten participants (8.4%) who received in-reach rehabilitation in their NCF died within 4 weeks of randomisation in this current study. This compares to a hospital-level risk adjusted 30 day mortality rate of 5.36% calculated from The UK National Hip Fracture Database (NHFD) (*n* = 3861) from 94 UK hospitals [27]. Our data suggest nursing home residents who had multiple co-morbidities and marked dementia appeared to respond favourably to medical and allied health intervention in this current study, at least while the rehabilitation was ongoing. However, once the rehabilitation was withdrawn at 4 weeks, their risk for death increased [4]. The combined medical, physiotherapy and dietetic interventions did reduce early mortality, but it is not known whether an extended program may have promoted sustained mortality benefits and ongoing functional improvements.

## Conclusion

Nursing home residents in this rehabilitation trial who received usual care received minimal physiotherapy after their return home. Those participants who received in-reach rehabilitation were able to tolerate higher levels of therapy and received 13 h of rehabilitation in the 4 weeks following their return home including a median of 161 min per week of physiotherapy compared to less than 30 min a week in the usual care group. However, perhaps more importantly, a change of culture in NCFs may have the greater effect, with the facility team coming together to set individualised goals for each resident with expert advice and support from the resident’s doctor, physiotherapist, dietician and NCF staff plus collaboration with hospital-based rehabilitation services. If NCF staff are able to provide a culture that promotes functional improvement, hip fracture will remain a major life threatening event, but some people may make mobility gains and potentially achieve an improved quality of life.

## Supplementary information

**Additional file 1: Table S1.** Additional usual care clinical services received by participants. **Table S2.** In reach rehabilitation provided after referral (nursing, speech pathology). **Table S3.** In-reach medical input from Geriatrician and Ortho-geriatric registrars. **Table S4.** In-reach physiotherapy received by participants. **Table S5.** In-reach dietetic intervention received by participants. **Table S6.** Adverse events for all participants according to group allocation. **Table S7.** Participants allocated to Intervention who died within 4 weeks of randomisation.

## Data Availability

All data generated or analysed during this study are included in this published article [and its supplementary information files].

## Abbreviations

NCF: Nursing Care Facility
