# Supplementary material for: People living in nursing care facilities who are ambulant and fracture their hips: description of usual care and an alternative rehabilitation pathway
Source: BMC Geriatr. 2020 Apr 9;20:128. doi: 10.1186/s12877-019-1321-x (PMC7147061; doi:10.1186/s12877-019-1321-x)
Supplement: Supplementary file 1 — Additional file 1: Table S1. Additional usual care clinical services received by participants. Table S2. In reach rehabilitation provided after referral (nursing, speech pathology). Table S3. In-reach medical input from Geriatrician and Ortho-geriatric registrars. Table S4. In-reach physiotherapy received by participants. Table S5. In-reach dietetic intervention received by participants. Table S6. Adverse events for all participants according to group allocation. Table S7. Participants allocated to Intervention who died within 4 weeks of randomisation. [file 12877_2019_1321_MOESM1_ESM.docx]

Table S1: Additional usual care clinical services received by participants

Table S2: In reach rehabilitation provided after referral (nursing, speech pathology)

| **Rehabilitation provided (hours)**  **Nursing**  *Received nursing review*  Face to face  Received follow-up review  Face to face  Received further follow-up review  Face to face  **Speech Pathology**  *Received swallow assessment*  Face to face | 13  13  8  8  5  5  5  5 |
| --- | --- |

Table S3: In-reach medical input from Geriatrician and Ortho-geriatric registrars

|  | Indication | No. of Ps | % of Ps |
| --- | --- | --- | --- |
| Education of patient/family regarding fracture, repair and complications | Confirmation and clarification of the nature of the injury, the surgical repair performed and any potential or actual complications suffered, often using copies of pre- and post-surgical radiological images | 92 | 77 |
| Review of medical conditions | Part of intervention protocol. | 111 | 93 |
| Review of medications | Part of intervention protocol. | 112 | 94 |
| Suggested changes to medications | Often related to analgesia, bowel management and bone protection.  Cessation of unnecessary medications following standard rational de-prescribing practice | 105 | 88 |
| VTE prophylaxis | Some patients on long-term anticoagulation, so not required.  Differences in protocol between acute sites and home rehabilitation service. | 74 | 62 |
| Review of pain * | Pain not managed with hospital discharge medications.  Working with therapists to achieve good pain cover while maintaining ability to engage in therapy. | 110 | 92 |
| Review of cognition including dementia/delirium | Most patients had long established dementia.  Early discharge home often meant patients showed signs of delirium on return to nursing home. | 93 | 78 |
| Physical examination, including review of observations | Part of intervention protocol. | 110 | 92 |
| Falls risk | Blood pressure issues. | 88 | 74 |
| Osteoporosis management | Vitamin D as the standard of care in this population.  If reasonably mobile and life expectancy >12 months, consider weekly oral bisphosphonates or alternative antiresorptive. | 87 | 73 |
| Skin care and pressure management | May refer to trial nurse, if rehabilitation team unable to ensure skin integrity. | 34 | 29 |
| Mood | Queries from family.  Review whether on antidepressant medications. | 77 | 65 |
| Continence | Queries from family, nursing home staff or trial intervention team. | 36 | 30 |
| Bowel management | Often related to use of opiates for pain management. | 78 | 66 |
| Nutrition and hydration | Reinforce dietician recommendations. | 63 | 53 |
| Red flags (indicators of deterioration suggested need for medical intervention) | As requested by nursing home, family and trial team. | 1 | 1 |
| Decision-making capacity | When advanced care plans not already in place. | 5 | 4 |
| End of life planning, advanced directives | Clarification of plan if required eg if get sick again, do they wish to be transferred to hospital. | 62 | 52 |

*All patients were initially prescribed regular paracetamol (1g orally qid [4times a day] or equivalent). “As required” oral opioids (eg oxycodone [Endone®] tablets 2.5-5mg 4 hourly) were used as breakthrough analgesia, particularly for incident pain associated with physiotherapy, mobilisation or hygiene interventions, given at a time that anticipated the pain. For those participants requiring frequent “as required” opioids, regular slow release oral or topical opioids (eg modified release oxycodone (OxyContin®) tablets, buprenorphine [Norspan®] patches) were used in the lowest dose required, guided by the daily “as required” use, especially if feedback from the trial physiotherapists suggested pain was a barrier to progressing the participant’s mobility.

Table S4: In-reach physiotherapy received by participants

|  | Indication | Type | No. of Ps | % of P’s |
| --- | --- | --- | --- | --- |
| Bed exercises | Early therapy when present high burden.  Limited functional antigravity therapy possible. | Active (assisted-resisted) limb exercises.  Bridging and moving in bed.  Functional bed transfers eg (using bedpan). | 95 | 80 |
| Chair exercises | Able to maintain sitting (supported-independent).  Spending increasing time in sitting. | Active (assisted-resisted) limb exercises.  Quadriceps exercises priority. | 106 | 89 |
| Standing exercises | Able to stand with 1-2 people and aides (wall bar or frame). | Standing at wall bar or frame.  Maintain static balance.  Maintain dynamic balance (reaching out of base of support, move limbs in standing)  Stepping all directions.  ***Using implicit learning system mainly.*** | 76 | 64 |
| Standing transfer practice | Able to stand with/without support.  Able to move to and from standing position with support. | Sit to stand transfer (start with stand lifter if required, then physical assistance of 1-2 people if necessary).  Stepping to turn (with/without assistance).  Stand to sit (with/without assistance) | 110 | 92 |
| Balance exercises (Otago exercises*) | When patient could understand and follow instructions and maintain safety. | Weight shift exercises.  Calf raises.  Toe raises.  ***Using explicit learning system.*** | 13 | 11 |
| Resistance exercises | When patient could tolerate and follow instruction/gesture. | Cuffed weights on wrists (resisted shoulder, elbow, wrist exercises).  Cuffed weights on ankles (resisted hip, knee and ankle exercises).  In particular resisted biceps-triceps and knee extension. | 44 | 37 |
| Mobilised | When patient capable of more than few steps safely. | Walking (standby-1 person-2 person assist) with frame to toilet, lunchroom, garden, around ward. | 96 | 81 |
| Stretches/PROM | When muscle and/or joint stiffness.  Inactive. | Limb exercises in bed. | 43 | 36 |
| Deep Breathing & Coughing | Signs or symptoms lung disease | Deep breathing exercises followed by coughing/huffing. | 4 | 3 |
| Identification of new issues | Issues identified including pressure areas, discomfort, inappropriate seating. | Review pressure aides eg cushioned heel supports.  Source pressure relieving mattress.  Review seating and provide recommendations, in particular pressure relieving seat cushion. | 16 | 13 |
| Education of nurses/carers | Specific education after identifying new issue. | Specific education to nursing home staff as any new issue arose eg ambivalence from resident, potential for skin integrity issues. | 23 | 19 |

* [9]

Table S5: In-reach dietetic intervention received by participants

| **Dietetics (initial visit) (n=107)** | Indication | Number of Ps who received | % of P’s |
| --- | --- | --- | --- |
| Feeding set up and assistance | Difficulty with self-feeding.  To develop Information for nursing staff handover sheet. | 68 | 64 |
| Fortnightly weigh/rescreen | High risk of malnourishment or diagnosed malnutrition on Subjective Global Assessment Score. | 46 | 43 |
| High protein/energy (HPE) diet and more intensive intervention | Supporting nursing home menu choices. Provision of nourishing high protein mid meals and “food fortification” strategies. | 58 | 54 |
| Modified texture | If swallow is compromised.  Dentition problematic. | 12 | 11 |
| Oral nutrition support | Prescription and supply Resource2.0 required or facilitation nursing home staff to supply similar if they prefer. May include additional supplements from nursing home range. | 63 | 59 |
| **Dietetics follow-up** |  |  |  |
| Feeding set up and assistance | As above | 18 | 17 |
| Fortnightly weigh/rescreen | As above | 25 | 23 |
| High protein/energy (HPE) diet | As above | 6 | 6 |
| Modified texture | As above | 0 | 0 |
| More intensive nutrition intervention | As above | 3 | 3 |
| Oral nutrition support | As above | 17 | 16 |
| Refer to local dietician | When need for follow-up beyond trial is anticipated. | 1 | 1 |
| **Dietetics 2^nd^ follow-up** |  |  |  |
| Fortnightly weigh/rescreen | As above | 1 | 1 |
| High protein/energy (HPE) diet | As above | 2 | 2 |
| More intensive nutrition intervention | As above | 4 | 4 |
| Oral nutrition support | As above | 2 | 2 |

Table S6: Adverse events for all participants according to group allocation

Table S7: Participants allocated to Intervention who died within 4 weeks of randomisation

| Participant | Place of death | If hospital LOS | Days after randomisation | Cause of Death * |
| --- | --- | --- | --- | --- |
| 1 | Acute Hospital | 6 days | 11 | Acute pulmonary oedema (6 days)  Acute coronary syndrome (6 days) |
| 2 | Nursing Home | NA | 6 | Myocardial infarction  Pneumonia (2 weeks) |
| 3 | Nursing Home | NA | 21 | Cardiac failure  Renal failure |
| 4 | Hospital | 3 days | 26 | Sepsis (1 week)  Pneumonia (1 week)  Electrolyte disturbance |
| 5 | Nursing Home | NA | 21 | End stage dementia (years) |
| 6 | Nursing Home | NA | 13 | Cardiac failure  Renal failure |
| 7 | Nursing Home | NA | 9 | Pneumonia |
| 8 | Nursing Home | NA | 16 | Pneumonia  Aspiration Pneumonitis |
| 9 | Nursing Home | NA | 28 | Chest infection  Chronic renal failure |
| 10 | Hospital | 1 day | 6 | Pneumonia  Respiratory Sepsis |

*Cause of death according to death registry.
